# Prevalence and factors associated with use of herbal medicine among women attending an infertility clinic in Uganda

**DOI:** 10.1186/1472-6882-14-27

**Published:** 2014-01-16

**Authors:** Henry Francisco Kaadaaga, Judith Ajeani, Sam Ononge, Paul E Alele, Noeline Nakasujja, Yukari C Manabe, Othman Kakaire

**Affiliations:** 1 Department of Obstetrics and Gynecology, Hoima regional Referral Hospital, Hoima, Uganda; 2 Department of Obstetrics and Gynecology, Makerere University College of Health Sciences, Kampala, Uganda; 3 Department of Pharmacology, Mbarara University of Science & Technology, Mbarara, Uganda; 4 Department of Psychiatry, School of Medicine Makerere University College of Health Sciences, Kampala, Uganda; 5 Division of Infectious Diseases, Department of Medicine, Johns Hopkins University, Baltimore, MD, USA

**Keywords:** Herbal medicine, Infertility, Uganda Sub-Saharan Africa, Traditional medicine

## Abstract

**Background:**

Infertility is a public health problem associated with devastating psychosocial consequences. In countries where infertility care is difficult to access, women turn to herbal medicines to achieve parenthood. The aim of this study was to determine the prevalence and factors associated with herbal medicine use by women attending the infertility clinic.

**Methods:**

This was a cross-sectional study of 260 women attending the infertility clinic at Mulago hospital. The interviewer administered questionnaire comprised socio-demographic characteristics, infertility-related aspects and information on herbal medicine use. The main outcome measure was herbal medicines use for infertility treatment. Determinants of herbal medicine use were assessed using multivariable logistic regression.

**Results:**

The majority (76.2%) of respondents had used herbal medicines for infertility treatment. The mean age of the participants was 28.3 years ± 5.5. Over 80% were married, 59.6% had secondary infertility and 2/3 of the married participants were in monogamous unions. In a multivariable model, the variables that were independently associated with increased use of herbal medicine among infertile patients were being married (OR 2.55, CI 1.24-5.24), never conceived (OR 4.08 CI 1.86-8.96) and infertility for less than 3 years (OR 3.52 CI 1.51-8.821). Factors that were associated with less use of herbal medicine among infertile women were being aged 30 years or less (OR 0.18 CI 0.07-0.46), primary and no education (OR 0.12 CI 0.05-0.46) and living with partner for less than three years (OR 0.39 CI 0.16-0.93).

**Conclusions:**

The prevalence of herbal medicine use among women attending the infertility clinic was 76.2%. Herbal medicine use was associated with the participants’ age, level of education, marital status, infertility duration, nulliparity, and duration of marriage. Medical care was often delayed and the majority of the participants did not disclose use of herbal medicines to the attending physician. Health professionals should enquire about use of herbal medicines. This may help in educating the patients about the health risks of using herbal medicine and may reduce delays in seeking appropriate care. Collaboration of health professionals with herbal medicine practitioners would help identify the common herbal medicines used for infertility treatment, their potential benefits and harm.

## Background

Infertility is a public health problem in middle and low-resource countries but continues to receive little attention [1]. In many cultures, having children is an essential part of life while infertility is seen in much of Sub-Saharan Africa (SSA) as a personal tragedy, with the potential to impact the entire family or community [1]. It is a dreaded condition that is associated with devastating psychosocial consequences [2-5]. In developing countries, where child-bearing is greatly valued, infertile couples are faced with problems ranging from overt ostracism or divorce to more subtle forms of social stigma leading to isolation and mental distress [4,6-8].

Child bearing is not only a continuation of one’s lineage but also economic security. In resource limited settings (RLS) where social security systems are non-existent, parents completely rely on their children for support as they age [9]. Thus married couples are under enormous pressure to procreate [4]. There are many barriers to effective and affordable biomedical infertility care in RLS like Uganda that have poor reproductive health indicator*s.* Infertility treatment and resources are lacking in the formal health sector due more urgent, life-threatening public health issues like maternal mortality [10] and the unmet need for infertility treatment remains large [6]. As a result, biomedical practitioners are often consulted later when religious and traditional methods have failed to provide a solution to the infertility problem [2,11].

Herbal medicine is widely used and a rapidly growing health system of economic importance. In Africa up to 80% of the population uses traditional medicine to help meet their health care needs [12]. In Asian countries such as China, traditional medicine accounts for around 40% of all health care delivered [12]. The prevalence of the use and utilization of traditional medicine in Uganda is similar to other parts of Sub-Saharan Africa due to its accessibility and affordability [13]. Traditional Herbal medicines, plants and shrubs extracts, are used to treat various ailments in Uganda. These medicinal plant concoctions are a key component of the primary health care system in Uganda especially in the rural areas [14]. Traditional herbal medicine use has been associated with severe and sometimes fatal complications [15,16]. However, many infertile women will take on considerable risk to conceive in order to avoid the social and psychological problems associated with childlessness [9].

The aim of this study was to determine the prevalence and factors associated with the use of traditional herbal medicines among patients presenting to the infertility clinic at Mulago Hospital. Due to the accessibility of traditional medicine, we hypothesized that patients may try herbal medicine first and delay accessing medical clinics.

## Methods

### Design and setting

This was a descriptive cross-sectional study performed at the infertility clinic of Mulago a national referral and university teaching hospital in Kampala, Uganda, between December 2011 and April 2012. On average, 50 patients attend the weekly infertility clinic. Forty percent of them attending for the first time. The majority (over 95%) of the patients attending the infertility clinic are females. All newly enrolled clinic patients were consecutively enrolled into the study until the sample size was achieved. According to a study in United Kingdom (UK) by Coulson and Jenkins [17] 40% of the infertile women had ever used complementary and alternative medicine for treatment of infertility. Using a 5% significance level and power of 90%, 260 women attending infertility clinic were enrolled into the study for us to establish the prevalence of Herbal medicine use.

### Data collection

The instrument for data collection (Additional file 1) was pretested among 15 patients attending the infertility clinic one month prior to the commencement of the study. An interviewer-administered structured questionnaire was used to collect the data. The purpose of the study was explained to the patients, and those who consented were interviewed by HFK, JA, OK and trained research assistants. Data was collected in a face-to-face interview. The questionnaire consisted of a demographic and fertility history and a history of use of herbal medicines for the treatment of infertility in the last 12 months prior to attending the infertility clinic. The participant was considered to have used herbs for treatment of infertility if she had swallowed or applied herbal medicines to part of her body. The data was double entered in Epidata version 3.1, cleaned and checked for completeness and exported to Stata 12 (Texas 77845, USA) [18] for analysis.

### Statistical analysis

Descriptive statistics (minimum, maximum, mean, and standard deviation) were applied for continuous variables and simple percentages for categorical variables univariable, bivariable (Table 1) and multivariable logistic regression analysis (Table 2) were used to identify factors which were independently associated with use of herbal medicines. Variables at univariate analysis that had p-value ≤ 0.2 were entered into a multivariable logistic regression model to determine factors that were independently associated with herbal medicine use. Variables that had P value ≤ 0.05 were considered statistically significant (95% confidence level).

**Table 1:** D**emographic and reproductive health characteristics of patients attending the infertility clinic at Mulago hospital**

**C** **haracteristics**	**Ever used herbs n = 198 (%)**	**Never used herbs n = 62 (%)**	**Totals n = 260 (%)**	**Crude OR/95% CI**	**P–value**
Age (years)					
≤30years	158 (78.8%)	34 (54.8)	192 (73.8)	1	<0.01
>30 years	40 (20.2%)	28 (45.2)	68 (26.2)	0.31 (0.17 - 0.57)	
Education level					
≤ primary	93 (47.0)	12 (19.4)	105 (40.4)	1	<0.01
≥ secondary	105 (53.0)	50 (80.6)	155 (59.6)	0.27 (0.14 - 0 .54)	
Religion					
Protestant	78 (39.4)	18 (29.0)	96 (36.9)	1	
Catholic	46 (23.2)	30 (48.4)	76 (29.2)	0.35 (0.18 - 0.71)	0.003
Moslem	53 (26.8)	11 (17.7)	64 (24.6)	1.11 (0.47 - 2.54)	0.802
Born again	21 (10.6)	3 (4.9)	24 (9.3)	1.625 (0.43- 6.01)	0.474
Marital status					
Not married	30 (15.2)	23 (37.1)	53 (20.4)	1	<0.01
Single	168 (88.8)	39 (62.9)	207 (79.6)	3.30 (1.73- 6.30)	
Type of marriage*					
Monogamous	95 (56.6)	27 (69.2)	122 (58.9)	1	0.15
Polygamous	73 (43.4)	12 (30.8)	85 (41.1)	1.73 (.82- 3.64)	
Ever conceived					
No (1º infertility)	53 (26.8)	35 (56.5)	88 (33.8)	1	<0.01
Yes (2º infertility	145 (73.2)	27 (43.5)	172 (72.2)	3.55 (1.96- 6.41)	
Infertility duration (in Years)					
1–3 years	72 (36.4)	30 (48.4)	102 (39.2)	1	0.09
> 3 years	126 (63.6)	32 (51.6)	158 (60.8)	1.64 (.92- 2.92)	
Duration with partner (in years)**					
1–3 years	91 (46.9)	20 (37.0)	111 (42.7)	1	0.20
> 3 years	103 (53.1)	30 (63.0)	133 (57.3)	1.64 (.92- 2.92)	
Distance from hospital (in KM)					
≤5 km	81 (40.9)	18 (29.0)	99 ( 38.1)	1	
6-10 km	72 (36.4)	28 (45.2)	100 (38.5)	0.57 (0.29 - 1.12)	0.103
>11 km	45 (22.7)	16 (25.8)	61 (23.4)	0.63 (0.29 - 1.34)	0.229
Ever used herbs for other health problem					
Yes	143 (72.2)	40 (64.5)	183 ( 70.4)	1	0.25
No	55 (27.8)	22 (35.5)	77 ( 29.6)	0.70 (0.38-1.28)	
Changed partner					
Yes	134 (67.7)	38 (61.3)	172 (66.2)	1	0.35
No	64 (32.3)	24 (38.7)	88 (33.8)	0.76 (0.42 - 1.37)	
Herbal use disclosure					
Yes	68 (34.3)	26(41.9)	94 (36.2)	1	0.28
No	130 (65.7)	36 (58,1)	166 (63.8)	0.72 (0.40-1.23)	

*Only married women answered this question.

**16 women were divorced were not currently in any form of relationship.

Km means Kilometers.

OR means Odds ratios.

CI Means Confidence intervals.

**Table 2:** Factors independently associated with herbal use among patients attending the infertility clinic at Mulago Hospital

**Variable**	**Adjusted OR (95% CI)**	**P- value**
Age (<30 versus > 30 years)	0.18 (0.07-0.46)	<0.001
Education level		
(< primary versus > secondary)	0.12 (0.05-0 .32)	<0.001
Marital status		
Married versus Single	2.55 (1.24 5.24)	0.011
Ever conceived		
No versus Yes	4.08 (1.86 - 8.96)	<0.001
Duration of stay with partner		
(1 – 3 years versus >3 years)	0.39 (0.16 – 0.93)	0.03
Duration of infertility		
(1 – 3 years versus >3 years)	3.52 (1.51 -8.21)	0.004

### Ethical consideration

The study was approved by the department of Obstetrics and Gynecology Mulago hospital, School of Medicine Research and Ethics Committee and Uganda National Council for Science and Technology. The participants gave written informed consent before enrollment into the study. The names and addresses of participants were not included to maintain confidentiality and to elicit an honest response.

## Results

Out of the 280 patients approached, 260 agreed to participate in the study (response rate: 92.9%). The main reason for refusal to participate in the study was lack of time for the interview. Among the 260 participants attending the infertility clinic, 76.2% had used herbal medicines for treatment of infertility. The mean age of the participants was 28.3 years ± 5.5, 80% were married, 66% had secondary infertility and 58% of the married participants were in monogamous unions. The majority (73.2%) had used herbal medicines in the last 12 months prior to seeking biomedical care and 63.8% did not disclose the use of herbal medicines to the attending physician. Adjusted multivariable analyses are shown in Table 2. The variables that were independently associated with increased use of herbal medicine among infertile patients included being married (OR 2.55, CI 1.24-5.24), nullparity (OR 4.08 CI 1.86-8.96) and those with infertility for less 3 years (OR 3.52 CI 1.51-8.821). However, factors that were associated with less use of herbal medicine among infertile couples were being 30 year or less (OR 0.18 CI 0.07-0.46), primary and no education (OR 0.12 CI 0.05-0.46) and living with partner for less than three years (OR 0.39 CI 0.16-0.93).

## Discussion

This study found that 76.2% of the participants had used herbal medicines to treat their infertility prior to seeking care at a medical clinic. These findings are lower than the estimates in a Turkish study where complementary and alternative medicines (CAM) were utilized by 82% of the infertile women [19]. However, it is higher than the 66% reported by Stankiewicz et.al among patients attending a reproductive health unit in Australia [20]. In Africa, traditional medical practice is a key resource for information, coping and herbal medication for health problems. The World Health Organization (WHO) estimates that use of herbal medicines on the African continent is widespread and prevalent [12]. The use of CAM by infertile patients is high worldwide [20,21]. There is a growing body of evidence that shows that a significant number of women or couples rely on CAM to enhance fertility or treat infertility [11,17,20-28]. Globally, the use of CAM by infertile couples or women varies considerably from 12% [26] to 91% [24]. A study among Lebanese women seeking infertility treatment showed that 41% of the women used CAM [23]. Similarly a study in the UK found out that 40% of 400 infertile women had used CAM as a therapy for infertility treatment [17]. However, in the United States (US) only 17% of infertile couples use herbal medicines [22].

We found a higher prevalence of herbal medicine use by women who were married and those who had never conceived. However, women who were less than 30 years of age and those who were less educated were less likely to use herbal medicines. Women who are educated are more likely to have some income that they use to pay for herbal medical treatment. Several studies have showed that older women, who are better-educated and employed with high incomes, are more likely to use CAM [20,22,24,29,30]. Addo and colleagues in Ghana, however, found that under privileged women attending the gynecological clinic were more likely to utilize herbal medications in the management of their infertility [27].

Our study shows that patients with infertility presented late to medical doctors for care. The majority of the participants presented after more than three years of infertility and did not disclose the use of herbs to the attending physician. This finding was similar to a study in Lebanon that showed that disclosure of CAM use to the attending physicians was low [23].

The participants who had been staying with a partner for more than three years were four times more likely to use herbal medications. The purpose of marriage in the African setting is procreation [2] and if a woman fails to conceive soon after marriage, couples seek for help and some opt for traditional herbal medicines as a solution [31]. The majority of the participants sought biomedical care for infertility after three years of failing to achieve a pregnancy. This could have been due to the lack of comprehensive infertility treatment in the mainstream public health care system in Uganda. Thus, women tried traditional medical practitioners first and it is only when they failed to conceive that they searched for the appropriate medical clinics. However, it is also known that the success of biomedical infertility treatment decreases with longer duration of infertility [32,33] and use of herbal medicines may reduce chances of conception [34]. Herbal medicines should be used with caution since they may worsen the infertility problem [35,36]. Evidence shows that phytoestrogens present in herbal medications exert negative estrogenic effects on implantation [35,36], adversely affecting pregnancy outcomes of biomedical treatment. Concurrent use of CAM and modern medical treatment for infertility has been shown to decrease pregnancy and live birth rates [37].

Our study acknowledges the following limitations. First, recruitment of participants took place in an infertility clinic at a national referral hospital which might have led to an underestimation of the prevalence of herbal medicine use since surveyed subjects have a potential bias toward conventional treatment. Second, given that participants were recruited from only one clinical setting, the results of the study may not be generalizable to the general population. However, the infertility clinic at Mulago national referral is the largest unit for infertility treatment in Uganda and is a major referral center for the treatment of infertility. The patients who are seen in this clinic are from different social and cultural background and were at different levels of infertility care. The results could be representative of the whole spectrum of the infertility problem in Uganda. Thirdly, this was a cross sectional study which does not allow us to establish causality between the various correlates and herbal medical use. Finally, the possibility of a recall bias cannot be ruled out in self-reports concerning herbal medical use.

## Conclusion

This study shows that 76.2% of the women attending the infertility clinic had used herbal medicines prior to seeking bio-medical care. Herbal medicine use was associated with the women’s age, level of education, marital status, duration of infertility, prior history of conception, and duration of stay with the spouse. At present, health care professionals do not routinely enquire about the use of herbal medicines. It is important that health professionals enquire from the patients about past or current use of herbal medicines. This may help in educating the patients about the health risks of using herbal medicine and may reduce delays in seeking appropriate care. Collaboration of health professionals with herbal medicine practitioners would help identify the common herbal medicines used for infertility treatment, their potential benefits and harm.

## Competing interests

The authors declare that they have no competing interests.

## Authors’ contributions

HFK, OK, JA conceived the study; HFK,JA, OK carried out the data collection; SO and OK analyzed the data; OK wrote the first draft; All authors contributed to the manuscript, read and approved the final version.

## Pre-publication history

The pre-publication history for this paper can be accessed here:

http://www.biomedcentral.com/1472-6882/14/27/prepub

## Supplementary Material

Additional file 1 Study instrument. Click here for file
