# Supplementary material for: Prevalence and factors associated with use of herbal medicine among women attending an infertility clinic in Uganda
Source: BMC Complement Altern Med. 2014 Jan 16;14:27. doi: 10.1186/1472-6882-14-27 (PMC3898407; doi:10.1186/1472-6882-14-27)
Supplement: Additional file 1 — Study instrument. [file 1472-6882-14-27-S1.doc]

# Additional file 1: STUDY INSTRUMENT

QUESTIONNAIRE FOR PREVALENCE AND FACTORS ASSOCIATED WITH USE OF TRADITIONAL HERBAL MEDICINE FOR TREATMENT OF INFERTILITY.

**GENERAL INFORMATION**

1. Study no: ___________________Date of interview ______________________

**Socio-demographic characteristics**

1. Age in completed years:________________________ Sex___________________
2. What is the highest level of education you have completed?___________________

No education Primary Secondary Post secondary

1. What is your tribe? …………………………………………………………….
2. What is your religion? Catholic Protestant Muslim Other ………….
3. What is your marital status? Single Married Separated

widowed Divorced No active relationship

1. Type of marriage Monogamous Polygamous
2. If polygamous how many wives do you have/ your husband have?..........................
3. How long have you stayed together?..........................................................................
4. Have you ever changed a partner? No .. Yes Why?..............................
5. What is the age of your current partner?.......................................................................
6. What is his /her education level?..................................................................................

No education Primary Secondary Post secondary

1. What is his/ her occupation?.............................................................................................
2. Does your partner have children from another relationship? Yes No
3. What type of house do you stay in Permanent Semi permanent
4. Do you or your family own?

Land Livestock Vehicle Motorcycle

Telephone Bicycle Television Radio Refrigerator

1. Who is the main income earner for your household? Self Patner Other
2. What is your occupation?....................................................................................................
3. How far do you stay from this hospital? < 5 kms 5-10 kms >10 kms

**General Health**

1. Have you had any health problems in the last 2 years

No Yes (specify)……………………………………………………

**Reproductive Health / Infertility**

1. Have you ever suffered from a reproductive health problem?

No Yes (specify)……………………………………………………

1. If yes did you seek any help?...........................................................................................
2. Where did you get help?

Hospital Clinic Herbalist Church Others

20. Have you ever conceived? Yes No (specify parity) …………………

1. What is the age of your last born? ………………………………………………………………
2. How often do you engage in sexual relations with your husband in a week?

None Once More than once

1. Have you suffered from any of the following from your spouse or immediate family?

Rejection Violence Stigmatize Sent away from home Other …………….

1. For how long have you known of this problem of infertility?......................................
2. What do you think is the underlying cause of your infertility?....................................

Witchcraft/Magic/Supernatural causes Curses Medical causes

1. Have you experienced any barriers in getting medical assistance of your infertility problem? No Yes (specify) …………………………………………………

**Herbal Medicine**

1. Have you ever used any form of herbal medicine for treatment of your condition in the last 12 months? No Yes
2. Have you ever accessed herbs from a friend or relative in the last 12 months

No Yes

1. Have ever visited a traditional healer or herbalist for assistance in the last 12 months?

No Yes

1. What are your reasons for using herbal medicine?......................................................
2. Do you know the type or composition of herbs given to you?

No Yes (specify) ………………………………………………….

1. How was the herbal medicine administered to you?

Orally Bathing Smoking Skin Incision

Smearing Others (specify) …………………………………………….

1. Have you experienced any benefits while using herbal medicines?

No Yes (specify) …………………………………………….

1. Have you experienced any side effects while suing herbal medicines?

No Yes (specify) …………………………………………….

1. Are you currently undergoing herbal medicine treatment? Yes No
2. Have you mentioned to any other health worker or doctor about the herbal medicine you used? Yes No why?....................................................................................

THANK YOU
